# Rhyl Sanitary Congress

**Published:** 1923-10

**Authors:** 


### RHYL SANITARY CONGRESS.

### SMOKE AND SCAVENGING.

THERE was a very large attendance at the Annual Conference of the Sanitary Inspectors' Association, which was held at Rhyl from Tuesday, September 4, to Friday, September 7. Mr. E. F. Moorhouse presided at the annual meeting of the Association on the Tuesday. The annual report stated that the membership was 1,731, an increase for the year of 56, which was considered to be highly satisfactory. The economy campaign was still raging, he said, and the Ministry of Health appeared to be supporting those authorities who were cutting down salaries—altogether inadequate for the responsible duties performed—and the Association was determined to ensure that adequate salaries were paid.

### The Housing Question.

In the evening Mr. W. Addington Willis gave an address on the Provision and Supervision of Houses and Dwellings. Mr. Willis said he could find nothing to account for the disastrous falling off of private enterprise in building houses, except the imposition of the Land Values Duties under the Finance Act of 1909-10. The imposition of the duties shattered the confidence of banks, insurance companies, and other mortgagees upon which private enterprise depended. The repeal of these duties in 1920 might improve the position, but financial confidence was almost as vulnerable as Humpty-Dumpty. Touching upon the present position as regards the supply of material for building, Mr. Willis said local authorities stood between the devil of profiteering, if prices were not controlled, and the deep sea of an insufficient supply of material if they were.

### A Gilbertian Situation.

The success of the present scheme, as foreshadowed by the 1923 Act, depended largely upon those who controlled labour, materials and money. Selfish obstruction by Labour, greed by manufacturers and vendors of materials, or restriction by financiers or investors, might easily bring to nought the newly devised plan. The country was paying out in unemployment doles, to 100,000 persons in the trades of building and constructive works, over £100,000 per week. This was equivalent, under the tenders of 1922, to the cost of 222 parlour and 264 non-parlour houses weekly. The spectacle of a nation in need of houses straining at the gnat of housing provision, and swallowing the camel of such a vast expenditure of non-productive money, would be Gilbertian if it were not tragic.

### The Presidential Address.

Sir William Collins delivered his Presidental Address on Wednesday. He said that the 800th anniversary of the celebration of the foundation of St. Bartholomew's Hospital had coincided with the justification and vindication of our voluntary hospital system as a glorious heritage of our race, and one which would not readily give place to any system of State maintenance. Reports on the question of health administration and housing were disappointing, yet the housing question stood more prominently related to the public health than any other matter of local government concern.

### Status of Inspectors.

Dealing with the status of the sanitary inspector, Sir William said he felt there should be created a body of examiners who were independent of the teachers of the candidates. It was also undesirable that in any new body any one institution should have a dominating influence, and he had yet to learn that the University of London might not play some useful part in any such examination board. The question of scale of salaries and the examination would require the careful and continued scrutiny of the council of the association.

### Smoke Abatement.

Mr. John Graham, M.A., Principal of Dalton Hall, University of Manchester, speaking on "Smoke and Noxious Vapours," said that this was not a good time for reform. The first thing needed in many cases was to alter or to add to plant, and at this moment of anxiety and uncertainty extra capital expenditure often could not be met. The Smoke Abatement Bill was a bad Bill containing one fatal provision in making universal the defence that "the best practical means have been used to prevent smoke, having regard to the cost and to local conditions and circumstances." That, however reasonable it sounded, hedged around offenders with a plea very expensive to meet, puzzling to magistrates, and discouraging to local authorities. The speaker urged that there should be one clear general regulation making smoke punishable as there was now. He believed they needed a body of skilled inspectors, independent of local bodies and empowered to prosecute as well as able to advise; but with the present skimping of all civilising expenditure that was the last thing the Ministry of Health would sanction. The abolition of smoke would save as much as the entire cost of the Navy, but to secure a part of those millions £10,000 was too much.

### Removal of Refuse.

An interesting paper on "House Refuse: Its Storage and Removal," was read by Mr. C. W. Laskey, Chief Sanitary Inspector for Eccles. The basis of all public health work was "scavenging and more scavenging." There was the need for a complete surveying and overhauling of the methods of storage and removal of house refuse before they could hope to bring about any real improvement. Mr. J. K. Crawshaw, M.B.E., Chief Sanitary Inspector for Lincoln, in his paper on "Pasteurized Milk," said pasteurization was not a policy of despair, but of commonsense. While it would take years to eliminate tuberculosis from the herds in the country, we could render harmless the tubercle bacillus in milk in half-an-hour at the cost of 2d. per gallon.

